# Bimanual Elbow Robotic Orthoses: Preliminary Investigations on an Impairment Force-Feedback Rehabilitation Method

**DOI:** 10.3389/fnhum.2015.00169

**Published:** 2015-03-30

**Authors:** Gil Herrnstadt, Nezam Alavi, Bubblepreet Kaur Randhawa, Lara A. Boyd, Carlo Menon

**Affiliations:** ^1^MENRVA Laboratory, School of Engineering Science, Simon Fraser University, Burnaby, BC, Canada; ^2^Brain Behavior Laboratory, Faculty of Medicine, University of British Columbia, Vancouver, BC, Canada

**Keywords:** exoskeleton, rehabilitation robot, stroke, bimanual, hemiparesis, cerebrovascular accident, neurorehabilitation, upper limb

## Abstract

Modern rehabilitation practices have begun integrating robots, recognizing their significant role in recovery. New and alternative stroke rehabilitation treatments are essential to enhance efficacy and mitigate associated health costs. Today’s robotic interventions can play a significant role in advancing rehabilitation. In addition, robots have an inherent ability to perform tasks accurately and reliably and are typically well suited to measure and quantify performance. Most rehabilitation strategies predominantly target activation of the paretic arm. However, bimanual upper-limb rehabilitation research suggests potential in enhancing functional recovery. Moreover, studies suggest that limb coordination and synchronization can improve treatment efficacy. In this preliminary study, we aimed to investigate and validate our user-driven bimanual system in a reduced intensity rehab practice. A bimanual wearable robotic device (BWRD) with a Master–Slave configuration for the elbow joint was developed to carry out the investigation. The BWRD incorporates position and force sensors for which respective control loops are implemented, and offers varying modes of operation ranging from passive to active training. The proposed system enables the perception of the movements, as well as the forces applied by the hemiparetic arm, with the non-hemiparetic arm. Eight participants with chronic unilateral stroke were recruited to participate in a total of three 1-h sessions per participant, delivered in a week. Participants underwent pre- and post-training functional assessments along with proprioceptive measures. The post-assessment was performed at the end of the last training session. The protocol was designed to engage the user in an assortment of static and dynamic arm matching and opposing tasks. The training incorporates force-feedback movements, force-feedback positioning, and force matching tasks with same and opposite direction movements. We are able to suggest identification of impairment patterns in the position-force plot results. In addition, we performed a proprioception evaluation with the system. We set out to design innovative and user immersive training tasks that utilize the BWRD capabilities, and we demonstrate that the subjects were able to cooperate and accomplish the protocol. We found that the Fugl–Meyer and Wolf Motor Function Test (pre to post) measured improvements (15 and 19%, respectively). Recognizing the brevity of the training, we focus our report primarily on the proprioception testing (32% significant improvement, *p*_prop_ = 0.033) and protocol distinctive features and results. This paper presents the electromechanical features and performance of the BWRD, the testing protocol, and the assessments utilized. Outcome measures and results are presented and demonstrate the successful application and operation of the system.

## Introduction

Caused by a neurological insult, stroke is the most common complex disability (Adamson et al., 2004), often accompanied by physical, cognitive, social, or psychological impediment (Kelly-Hayes et al., 2003). Stroke accounts for almost 10% of all death and is the second leading cause of death worldwide (Mathers et al., 2008). There were 6.8 million people with stroke in the United States in 2013 with about 800,000 stroke incidents per year (Go et al., 2014). The projected direct (medical expenses) and indirect (productivity loss) costs by 2030 in the United States are expected to be $95.6 and $44.4 billion, respectively (Heidenreich et al., 2011). There is therefore, a crucial need to mitigate associated monetary and health costs via better treatment and rehabilitation strategies.

Over 60% of stroke survivors suffer from some upper-limb impairment (Cauraugh et al., 2005). Somatosensory deficits account for 10–60% of these cases, with proprioception forming 34–64% of these deficits (Connell et al., 2008; Torre et al., 2013). Proprioception is defined as the sense of position, motion, and effort (Simo et al., 2014). Literature suggests that sensory and proprioceptive feedback is crucial for proper motor control (Goble et al., 2006; Vidoni and Boyd, 2008) and is linked to motor control learning (Rose et al., 2004; Crespo and Reinkensmeyer, 2008; Marchal-Crespo and Reinkensmeyer, 2009). Several clinical and automated procedures have been proposed in the literature to measure proprioceptive deficits, but these assessments have either poor reliability or ordinal classification (present or absent), without defining the extent of impairment (DeGowin and DeGowin, 1987; Lincoln et al., 1998; Winward et al., 2002; Leibowitz et al., 2008). Other scientific quantitative methods to assess proprioception involve mechanisms that measure limb positional errors (Goble and Brown, 2008; Leibowitz et al., 2008; Simo et al., 2014). There is a need of objective proprioceptive measurement tools that may be incorporated with regular therapy to provide rehabilitation and ongoing proprioceptive evaluation.

Mostly, stroke rehabilitation in the chronic phase is guided by a physical therapist to achieve maximal functional improvement. A few rehabilitation techniques that have shown promise in inducing neuroplasticity are constrained-induced therapy, impairment oriented-training (IOT), robotic interactive therapy, and virtual reality (VR) (Dobkin, 2004; Krakauer, 2006; Harvey, 2009). Data show that high bouts of intensive training with these methods coupled with distributed practice, task variability, bilateral symmetrical arm movements (Cauraugh and Summers, 2005), and robotic therapy (Riener et al., 2005) may help improve retention and maximize gains post-stroke (Krakauer, 2006). In particular, robots offer advantages such as consistency and precision that may aid in the design of unique bimanual rehabilitation protocols and may be programed to suit individual post-stroke needs. Indeed, robotic therapies, in recent years, have gained recognition as a viable rehabilitation tool (Reinkensmeyer et al., 2000; Cramer and Riley, 2008; Krebs et al., 2008; Staubli et al., 2009).

## Bimanual Robotic Rehabilitation

A number of robotic devices have been developed for bimanual rehabilitation, with varying degrees of freedom (DOF) and utilizing different bimanual training modalities ranging from passive to active movements and incorporating haptic feedback. In an earlier work by Lum et al., a 1-DOF, wrist flexion/extension bimanual device was developed to perform rhythmic transporting and squeezing tasks (Lum, 1993). A second work by Lum et al. presented the “Bimanual Lifting Rehabilitator” (Lum et al., 1995), a 2-DOF device helping to lift and replace an object off a table with two hands. Further, Burgar et al. summarized three generations of the mirror-image movement enabler (MIME) prototype development and clinical trials (Burgar et al., 2000), and Lum et al. presented a follow-up clinical study (Lum et al., 2006). The MIME robotic device was designed to train the shoulder and elbow, in a three-dimensional space, using a 6-DOF manipulator. A recent clinical study using the MIME shows equal or greater benefits compared to conventional therapy at the 6-month follow-up period (Burgar et al., 2011). Another study by Hesse et al. presented a 1-DOF bench-top computer-assisted training device used to train the forearm or wrist (Hesse et al., 2003). Three training modes were implemented: passive, active, and active with resistance. A similar design with the addition of force feedback to the unaffected hand was developed by Rashedi et al. (2009). Burgess et al. explored skill transfer of bimanual training by using a 2-DOF planar manipulandum device with a single handle grasped using both limbs (Burgess et al., 2007). The results provide evidence of similar skill transfer from bimanual training to both the dominant and non-dominant hands. Several recent papers compared robotic bilateral and unilateral training (and a control group) using several devices including the HapticMASTER (a 3-DOF manipulator), the Bi-Manu-Track system (a 1-DOF manipulator for the wrist or forearm), and the ARMin and EXO-UL7 (7-DOF exoskeletons) (Lewis and Perreault, 2009; Kim et al., 2013; Wu et al., 2013; Artemiadis, 2014). Rather than showing conclusive results, either method (bilateral and unilateral) tends to outperform the other by improving motor abilities in different performance metrics, criteria, and/or tasks.

Despite a number of studies comparing bimanual protocols, the results thus far are ambiguous and inconclusive (Lum et al., 2006; Summers et al., 2007; McCombe Waller et al., 2008; Richards et al., 2008; Coupar et al., 2010; Van Delden et al., 2012). However, through these studies, several important aspects of rehabilitation have been identified, including stroke severity, impairment level, haptic and proprioceptive feedback, workspace limitations (e.g., planar vs. 3D), unimanual vs. bimanual training, proximal or distal musculature training, and dosage (Rose et al., 2004; Stewart et al., 2006; Cauraugh et al., 2010; Latimer et al., 2010; Van Delden et al., 2012), that may affect the outcomes of the rehabilitation. Nevertheless, determining a highly efficacious practice, that can significantly advance rehabilitation, remains elusive.

In this preliminary study, we designed a user-driven bimanual elbow orthoses with the objective of increasing the user’s active role in the therapy. We hypothesized that bimanual rehabilitation, performed with the aid of a robotic system that enables the user to perceive with her/his non-hemiparetic arm, the movements as well as the forces applied by her/his hemiparetic arm can be used to successfully rehabilitate individuals with stroke and facilitate inter-hemispheric interactions (e.g., somatosensory). As a first step, the scope of the study was to appraise the suitability and to demonstrate the competence of the BWRD in carrying out the novel force-feedback-based methods and protocol, tested on individuals with stroke.

## Materials and Methods

### Overview

**Figure 1:**
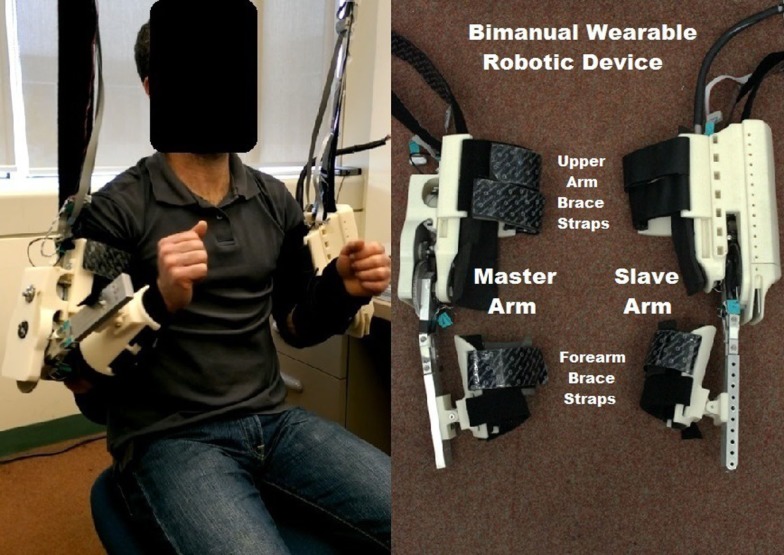
**BWRD system in use, and devices top view**.

The proposed bimanual wearable robotic device (BWRD) is composed of two 1-DOF robotic orthoses connected in a Master–Slave configuration. The BWRD is designed to train the movement of the elbow and is donned on both the non-hemiparetic (Master exoskeleton) and hemiparetic (Slave exoskeleton) arms as shown in Figure 1. Kinematic and force signals are processed and fed to the control algorithm.

The Master and the Slave are connected such that any movement of the Master induces a movement of the Slave. In turn, any resistance exerted by the hemiparetic arm on the Slave induces an equal resistance force on the non-hemiparetic arm through the Master.

### System architecture

The two fundamental aspects of the BWRD are: (1) to control the movement of the hemiparetic arm using the non-hemiparetic arm and (2) to send force feedback from the hemiparetic arm to the non-hemiparetic arm.

The BWRD Master and Slave devices are capable of producing movements in 1-DOF for the elbow in flexion/extension. Position and force sensors are embedded in the mechanical structure. The participant’s ability to control the impaired limb is an important additional safety feature, different from other robotic therapies in which the subject has none or limited control over their trained limb.

#### Hardware

The integrity of the structure was of particular interest in the design of the BWRD. A human elbow joint can generate torque of up to 70 Nm (Tsagarakis, 1999). The protocol tasks in the study were such that the user was expected (depending on their ability) to apply loads to the system. Therefore, a robust mechanical construction, utilizing aluminum and steel on key components, was conceived in order to withstand the applied forces.

**Figure 2:**
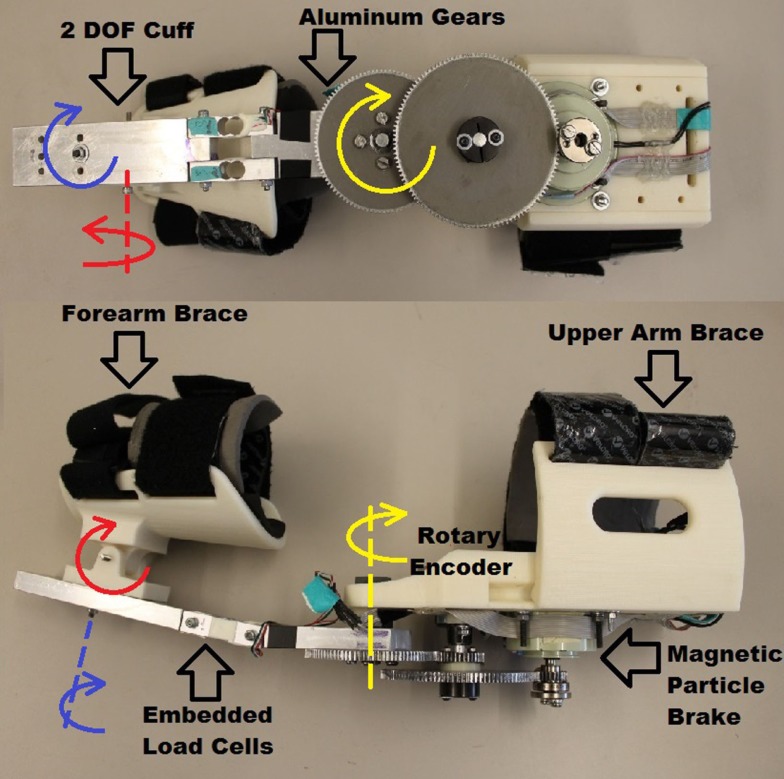
**Master device view of the electromechanical components and structural assembly**.

**Figure 3:**
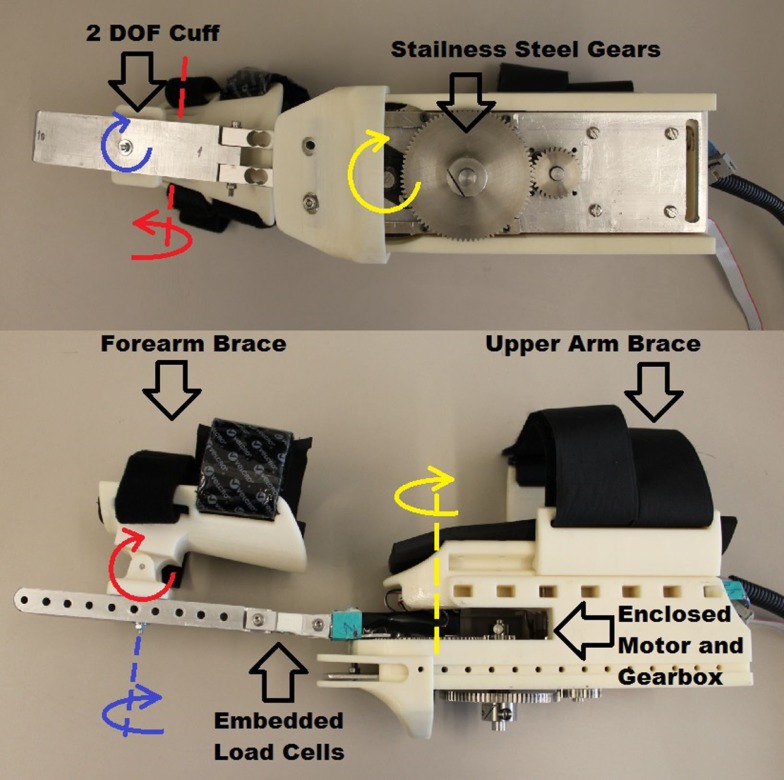
**Slave device view of the electromechanical components and structural assembly**.

In the BWRD, the actuator of the Master joint assists to control the speed of the movement by dampening the motion through a micro magnetic particle (magneto-rheological) brake (Chaintail ZKYS10AA). Motion can only be generated by the user’s own movement, contributing to the inherent safety of the system. The brake is powered by a 24 V battery pack. A custom back-drivable aluminum spur gear system, with a ratio of 13.9:1, was implemented resulting in a theoretical total torque output of the Master of 13.9 Nm. An encoder (CUI AMT10X series) is embedded to capture angular position. Table 1 lists the electromechanical properties of the brake. The brake is controlled using a MOSFET (IRFZ34N) with a pulse width modulation (PWM) signal from a NI data acquisition (DAQ) device. The actuation of the Slave is accomplished using a brushless DC motor (Maxon EC 45 flat 50 W). The Slave device is powered by a 36 V battery pack. Mechanical stops are in place to ensure the device does not actuate past set limits, in order to avoid injury. The total gear ratio of the Slave arm is 192:1, and is composed of a Maxon gearbox (Spur Gearhead GS 45) with a ratio of 18:1, and custom spur gears with a ratio of 10.7:1. The theoretical total continuous torque output of the Slave is 18.24 Nm. A Maxon encoder (Type-L, 256-CPT) is combined to capture angular position. Table 1 also lists the motor and gearbox properties. The motor is controlled using a Maxon servo controller (ESCON Module 50/5) and a NI DAQ. Phidget micro load cells (3133 – Micro load cell 0–5 kg) force sensors are embedded into the forearm structure of both the Master and Slave. The load cell signals are acquired using a Phidgets bridge amplifier (1046 – Phidget Bridge 4-Input) connected via USB to a PC. Mechanical assembly of the Master and Slave devices is shown in Figures 2 and 3, respectively. Adjustability and ergonomic features were incorporated in the design to improve fit and comfort. The upper arm cuffs are adjustable along the arm axis to accommodate for different body sizes. A 2-DOF pivoting forearm cuff has been implemented to improve fit and alignment as shown in Figures 2 and 3. The devices easily transform for either the left or right arm by swapping the sides of the upper arm braces.

**Table 1:** **Magnetic particle brake, Maxon motor, and gearbox characteristics**.

MR brake		EC 45 motor		Spur gearhead GS 45	
Rated torque (Nm)	1	Nominal voltage (V)	36	Reduction	18:01
Rated current (A)	0.42	Nominal torque (mNm)	90.5	Number of stages	3
Power (W)	10	Nominal current (A)	0.828	Weight (g)	224
Weight (kg)	0.54	Weight (g)	110	–	–
Diameter (mm)	56	–	–	–	–

The total weights of the Master and Slave arms are 1.9 and 3.0 kg, respectively. To mitigate the BWRD weight and avoid burdening the participants’ shoulders, the Master and Slave arms were supported by a custom aluminum structure (Bosch Rexroth Aluminum Structural Framing).

#### Software and control

**Figure 4:**
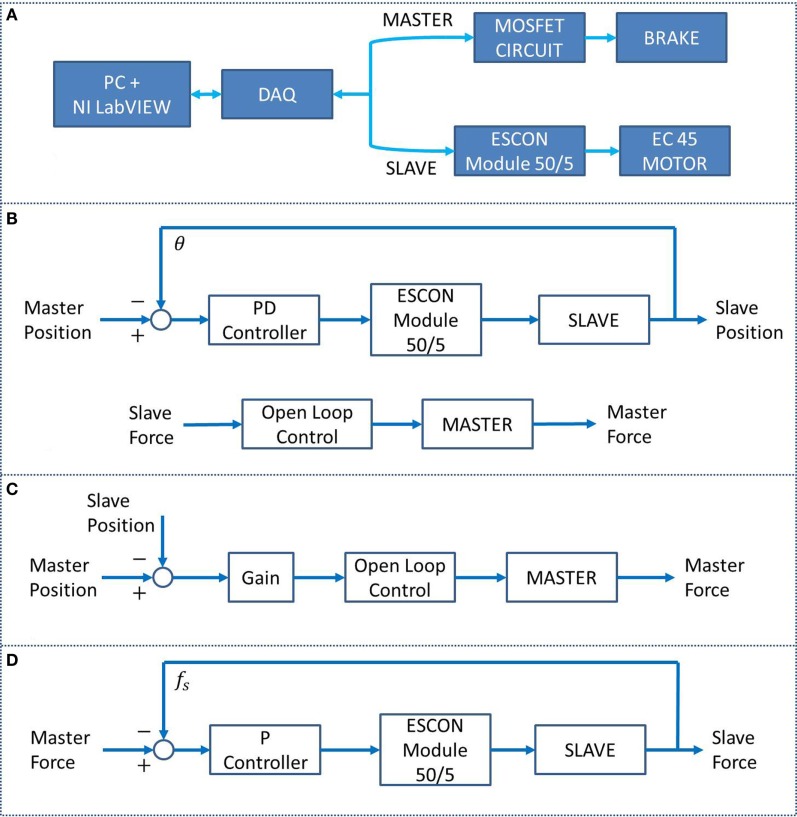
**(A)** BWRD system connectivity. **(B)** The PC–FC is a parallel position and force controller used in tasks #1 through #3 and #5. **(C)** The PEFC, used in task #4, generates a resistance force on the Master orthosis based on the position error between the Master and the Slave arms. **(D)** The FPVC, used in task #6, generates a movement of the Slave device with a velocity proportional to the forces applied against the Master and/or Slave devices.

An NI LabVIEW 2013 program was used to operate the BWRD system. Calibration data were acquired for the Master and Slave load cells, as well as for the brake. Figure 4A shows the BWRD component connectivity. In total, three control methods were used in the training protocol. First, a parallel design controller was implemented, incorporating a Position Controller and Force Controller (PC–FC). The PC–FC position sub-controller used a proportional-derivative (PD) structure in conjunction with an internal embedded (ESCON Module 50/5) open loop speed controller. The PC–FC position sub-controller translates a Master arm movement to a parallel movement in the Slave arm. The PC–FC open loop force sub-controller was implemented in the form of a quadratic equation, describing the brake’s torque–voltage relationship. The PC–FC force sub-controller projects forces from the Slave device to the Master device; forces applied with the arm against the Slave device are projected, such that the opposite arm encounters an equal force against the Master device. The PC–FC control method was used in the majority of the protocol tasks as shown in Figure 4B. Additionally, the PC–FC position and force sub-controllers were implemented to operate completely independently. The second controller, a position-error force controller (PEFC), was implemented as shown in Figure 4C. The PEFC generates a resistance force on the Master orthosis proportional to the position error between the Master and the Slave arms. The third controller, a force-proportional velocity controller (FPVC), was implemented as shown in Figure 4D. In this mode, a force applied by the user’s arms against the Master and/or Slave devices resulted in a movement of the Slave device, with a velocity profile proportional to the sum of the forces.

The load cell and main software loop sampling frequency were set to 24 and 20 Hz, respectively. A compensation algorithm was implemented on both the Master and Slave load cells to account for the gravitational forces. Consequently, only the user’s applied forces on the BWRD were read. A feed-forward compensation equation was implemented in the form of *T* = *M*sin(θ). *M* is the gravity torque of the orthoses elbow joint when in a horizontal forearm position, and θ is the elbow angle such that when the arm is vertical, there is no gravitational compensation. In addition, software constraints were set on each joint that redundantly prevent exceeding angular limits.

### Experimental protocol

We carried out a preliminary study for verification and validation of the BWRD and the chosen protocol. We were interested in the functional aspect of BWRD as it facilitated movement and execution of the custom exercises. We tested the suitability, viability, and the subjects’ acceptance of the system and the protocol in this first study. For this pilot study, we recruited eight participants with chronic stroke. All participants satisfied the inclusion criteria (as described in section “Participants’ Selection and Assessment”) and provided informed consent. Ethics approval for the study was obtained from the SFU Office of Research Ethics. One participant withdrew during the third day of testing due to stress and fatigue in the shoulder. Therefore, we present the data on seven participants only.

The assessment of the training protocol is discussed in the Section “Participants’ Selection and Assessment.” The physical training sessions are detailed in the Section “Training.” Participants completed three training sessions within a time span of a week.

#### Participants’ selection and assessment

Eight participants with chronic stroke (>6 months post-stroke) were recruited from the local community. Inclusion criteria included: between the ages of 35 and 85, with a single or multiple episodes of unilateral stroke and a score >24 on the Montreal Cognitive Assessment (MoCA). One study participant (P01) had expressive aphasia (15/30 on the Frenchay Aphasia Assessment) where he was unable to complete the MoCA test (Al-Khawaja et al., 1996). In addition, to avoid confounding changes, participants did not receive other forms of rehabilitation concurrent with the study. Participants were excluded from the study if they had a psychological diagnosis or any neuromuscular, skeletal, or cardiovascular conditions that could interfere in performing any of the experimental tasks. They were also excluded if they had a history of seizures/epilepsy, substance abuse, or head trauma. Participant information is presented in Table 2.

**Table 2:** **Participant data**.

Participant	Gender	Age	Time post-stroke (month)	Cognitive (MoCA)	FMA stroke score	Handedness (R/L)	Affected hand
P01	M	64	102	*	11 (severe)	R	R
P02	M	60	87	30	13 (severe)	R	L
P03	M	69	18	26	31 (moderate)	R	L
P04	M	67	35	25	27 (severe)	R	L
P05	M	82	35	26	22 (severe)	R	L
P06	M	66	42	26	63 (mild)	R	L
P07	M	78	14	25	30 (moderate)	R	L
Mean (SD)	–	69.4 (7.8)	47.6 (33.8)	–	28.1 (17.3)	–	–

*MoCA, Montreal Cognitive Assessment; SD, standard deviation; FMA stroke severity defined in Pang et al. (2006). FMA is scored out of a total of 66 points*.

*participant P01 had expressive aphasia (15/30 on the Frenchay Aphasia Assessment) where he was unable to complete the MoCA test (Al-Khawaja et al., 1996).

Pre and post-assessments included the Fugl–Meyer Assessment (FMA), the Wolf Motor Function Test (WMFT), and a proprioception evaluation for all the participants. Pre-assessments for all tests were done prior to session 1 and post-assessments were done after session 3 of the study. In addition, the proprioception data were collected after session 2 as well.

The proprioception assessment was performed as follows. The Slave device was donned on the impaired arm, brought to a predefined position and set mechanically to maintain that position (a passive target). Participants were asked to match the Slave arm position angle with the Master arm — contralateral matching. Position matching is a common approach to quantify proprioception and involves measuring joint position matching errors (Goble, 2010). Slave arm positions were assessed arbitrarily at approximately 30°, 50°, or 80°. The task was performed with eyes closed to prevent visual feedback and repeated twice for each angle (six repetitions per session). Position errors were consequently calculated.

#### Training

The training tasks phase involved proprioceptive and haptic feedback exercises, offering a variety of ways to train the paretic arm and assess its functionality. In the training protocol, we referred to the Master arm as the non-hemiparetic arm and the Slave arm as the hemiparetic arm. Time was allocated to don the devices, and then participants were instructed to cycle through tasks #1 through #6 twice. Five repetitions were performed for tasks #1 through #3 and #5 in each cycle (10 repetitions in each task in total). Two repetitions were performed for task #4 for three angles in each cycle (12 repetitions in total), and 2 repetitions were performed for task #6 for 3 masses in each cycle (12 repetitions in total). Breaks were provided between tasks and upon request. The tasks were stopped when the required number of repetitions was achieved, unless the subject needed to stop prematurely for any other reason. The physical training was completed approximately in 1 h. The training tasks are described within Table 3. For all the tasks, a fully extended elbow corresponds to a 0° angle.

**Table 3:** **Training tasks**.

Task #	Task name	Description	Repetitions per session	Control
1	Slave arm relaxed	Relax Slave arm and move the Master arm through the full range of motion (ROM), flex/extend.	5 × 2 cycles = 10	PC–FC
2	Slave arm follow	Move Master arm and follow (mirror movement) with the Slave arm.	5 × 2 cycles = 10	PC–FC
3	Slave arm dynamic resistance	Flex/extend elbow of the Master arm and follow with the Slave arm. During the movement, the Slave arm voluntarily increases its resistance to the movement.	5 × 2 cycles = 10	PC–FC
4	Feedback arm match	Set the Slave device to maintain an uninformed position. Ask the participant to move the Master arm along the full ROM and detect the point of minimum resistance, corresponding to the Slave arm position.	2 (for the 3 angles) × 2 cycles = 12	PEFC
5	Conditional arm dynamic	The Master arm attempts to initiate a movement. Motion is easily generated only if sufficient force is applied in the desired direction by the Slave arm (force mirroring). The result is movement mirroring.	5 × 2 cycles = 10	PC–FC
6	Conditional arm static	Master device is locked. Master arm pushes against it causing the Slave device to move in the force direction, and in proportional velocity. Push with Slave arm in opposite direction to maintain matching position.	2 (for the 3 angles) × 2 cycles = 12	FPVC

*The control column indicates the control method used in the respective task. PC–FC, parallel position and force controllers; PEFC, position-error force controller; FPVC, force-proportional velocity controller. The control methods are detailed in the Section “Software and Control”*.

The tasks performed can be broken down into three distinct groups A–C:

- Force-feedback movement – tasks #1–3. Affected arm interaction forces with the Slave device were fed back to the unaffected arm.
  - Task 1 – feedback to the Master arm should, in an ideal case, consist of a Slave arm gravity component only.
  - Task 2 – the purpose is to achieve an unrestricted Master arm movement (no resistance in the system, i.e., by not preceding or succeeding with the Slave arm).
  - Task 3 – the goal is for the participant to voluntarily control the resistance forces imposed by their Slave arm on the Master arm.
- Force-feedback positioning – task #4. Feedback related to the position of the affected arm was sent to the unaffected arm to guide and increase positioning accuracy.
  - Task 4 – angles were arbitrarily set to approximately 30°, 50°, or 75°. A 75° angle was used, different from the proprioception assessment, to allow more range of motion (ROM) to explore the force feedback.
- Force matching – tasks #5–6. These tasks required activating and applying equal force magnitudes by both arms simultaneously.
  - Task 5 – the goal for this task was to have equal magnitude and direction forces.
  - Task 6 – the goal for this task is to have equal magnitude and opposing forces such that the Slave arm maintains the same angle as the Master arm (for 3 s). This was implemented by fixing the Master arm and attaching weights (1, 2, and 4 lbs) to simulate the applied Master arm force.

## Results

Results were obtained from seven participants. Overall training was tolerated well by these participants and completed without adverse effects. Occasional subjective discomfort or pressure from the devices was addressed promptly. The data collected are presented in the following subsections “Task Performance, Motor Function Assessment Data (FMA and WMFT), and Proprioception Assessment Data.” The mean scores for all the tests — the FMA, WMFT, and proprioception — improved after session 3, as shown in the fourth column in Table 4. However, only the proprioception improvement was statistically significant. Positive numbers indicate an improvement.

**Table 4:** **Mean clinical and kinematic measures of all participants**.

Variable	Pre-treatment mean (SD)	Post-treatment mean (SD)	% Change
	(n = 7)	(n = 7)	
WMFT-time	32.0 (31.0)	25.9 (25.3)	19.1
FMA	28.1 (17.3)	32.4 (18.2)	15.2
Proprioception	46.2 (11.9)	31.4 (12.1)	32.8

*WMFT-time, Wolf Motor Function Test time performance (s); FMA, Fugl–Meyer Assessment; Proprioception, proprioception position matching errors (degrees); SD, standard deviation (degrees)*.

### Task performance

**Figure 5:**
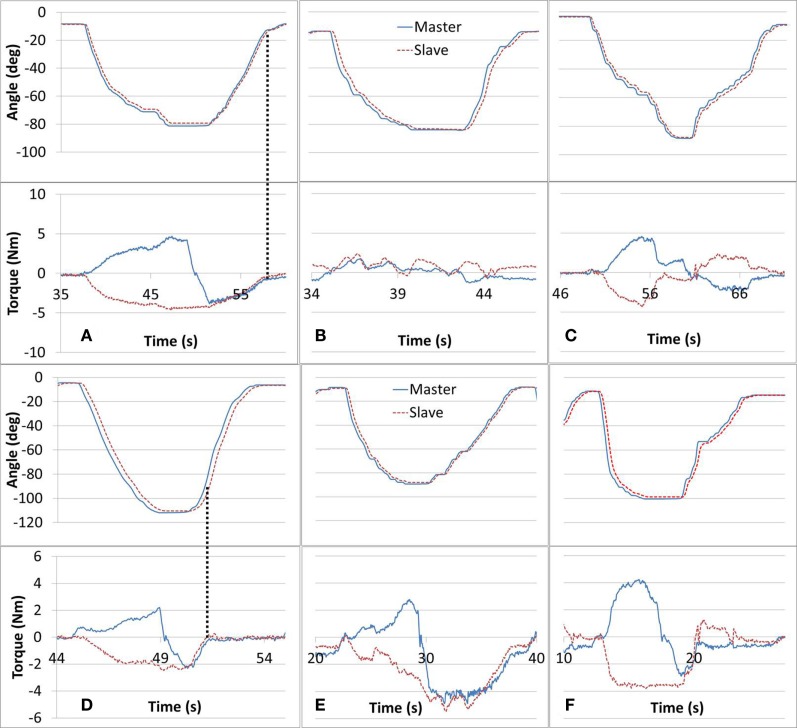
**Tasks #1, 2, and 3 intended performance is shown in (A–C), respectively**. Deficient performance of tasks #1 through #3 is shown in **(D–F)**, respectively. In task #1 **(A)**, the paretic arm is intended to be relaxed, in task #2 **(B)**, the paretic arm is intended to be active in the same direction with the intact arm motion, and in task #3 **(C)**, the paretic arm is intended to be active but opposing the motion of the intact arm.

The study protocol tasks targeted and challenged participants’ motor control and sensory capabilities. The haptic feedback provided added stimulus, designed to offer awareness of the affected arm and enhance the performance of the training tasks by exposing unintentional forces (e.g., caused by arm stiffness or spasticity). Tasks #1, #2, and #3 were designed to help with cognizance of the affected arm ability in dynamic exercises. Successful completion of tasks #1, #2, and #3 are shown in Figures 5A–C, respectively. In task #1, the affected arm was intended to be relaxed. In Figure 5A, the Slave force observed is composed of the forearm gravity component and possibly some involuntary muscle tone, while the Master arm force is alternating direction based on the direction of its movement. In task #2, the affected arm was intended to match the non-affected arm movement such that forces recorded were minimal. As shown in Figure 5B, both arms demonstrated low force levels indicating that voluntary mirror movements were performed independently, unassisted by the robot, by the participant’s musculoskeletal system. In Figure 5C, related to task #3, the participant’s affected arm was successful in dynamically applying forces in opposing directions, with comparable magnitudes in both directions. Figures 5A–C demonstrate the desired performance for tasks #1–3; however, not all the participants’ sessions were performed as successfully, likely due to arm weakness, spasticity, and limited proprioception or motor control. Some deficiencies in the completion of tasks #1–#3 are presented in Figures 5D–F, respectively. In Figure 5D, the participant was able to perform the elbow-flexion component of the movement as intended. However, as indicated by the vertical lines, in the elbow-extension component of the movement, the force that was applied by the affected arm dropped rapidly suggesting that the arm was not weighing down on the Slave device (perhaps not fully relaxed). In Figure 5E, the participant was unsuccessful in mirroring the movement with the affected arm resulting in increased resistance forces to the Slave device. These forces were consequently projected to the Master device and sensed by the unaffected arm. The intention in task #3 was to volitionally apply opposing forces. As shown in Figure 5F, the participant successfully resisted with the affected arm during the flexion component of the motion but the level of resistance was significantly reduced during the elbow-extension component.

**Figure 6:**
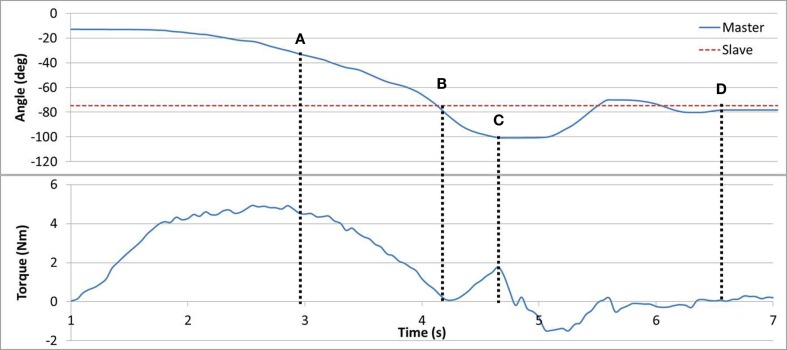
**Task #4**. In line (A), angular error and torque are jointly reducing. In line (B), no angular error results in zero torque. In line (C), angular error is increased triggering an increase in torque. In line (D), the subject has reached his target estimate.

In Task #4 subjects were asked to locate the point of minimum resistance using their Master arm. The brake provided resistance proportional to the angular position difference between the Master and the Slave devices. Subjects were asked to rely on the haptic feedback though vision was left unobscured. It was instructed that the point of minimum resistance corresponded to the opposite arm angle. The torque curve can be seen in Figure 6. In line (A), the resistance torque is reducing along with the position error until line (B), and then increasing as the position error increases up to line (C). Eventually, the subject reached the guessed target position in line (D). The algorithm eliminated resistance completely when the absolute angular error was within ±2°, which resulted in a range without haptic feedback. Subjects were requested to try and locate the middle of the haptic-less gap.

**Figure 7:**
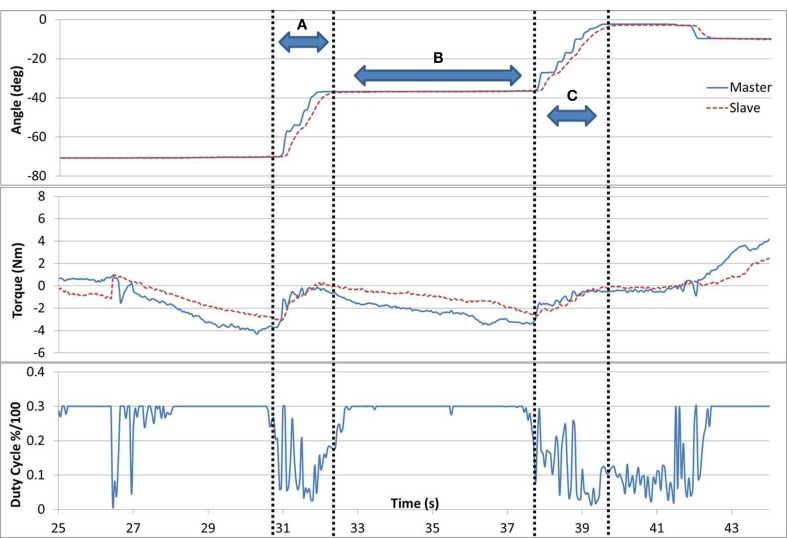
**Task #5**. In gaps (A) and (C), the subject is successfully applying equal forces with both arms resulting in a low resistance (duty cycle) and a parallel movement. In gap (B), the forces between the arms diverge resulting in higher resistance and reduced motion.

Successful completion of task #5 is shown in Figure 7. When the difference in forces between the arms was >1 Nm, the duty cycle was set to a constant maximum value. But, if the difference was <1 Nm, the brake duty cycle reduced proportionally. In gaps (A) and (C), the subject successfully applied equal forces with both arms (<1 Nm difference), resulting in a movement with low resistance (duty cycle). In gap (B), the force difference between the arms was increased, which resulted in higher resistance and reduced motion. A consequence of successful matching of the forces in task #5 can be seen as the forces naturally tended to 0, as observed in gaps (A) and (C). This is inherent to the task as when forces are matching, movement is uninhibited and therefore the interaction force between the arm and the device decreases.

**Figure 8:**
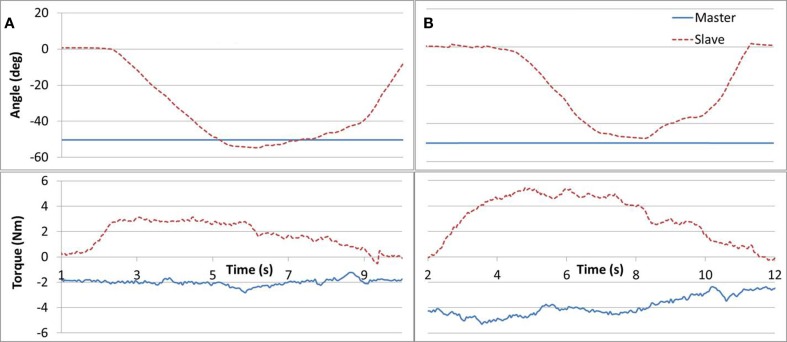
**Task #6**. Subfigures **(A,B)** refer to a 2 and 4 lb cases, respectively. A Master force drift can be observed in **(B)**.

Task #6 required the subjects to apply equal force magnitudes (similar to task #5) but in opposite directions in order to maintain a static position. The task was implemented as follows. The Master arm was positioned at 50° angle and fixed by locking the brake. Three different loads (1, 2, and 4 lb.) were then attached one at a time to the Master arm. The subject was requested to relax his unaffected arm while applying upward force with the affected arm, until their angles matched and to hold the position for 3 s. The task required that the affected arm equaled the gravity force measured in the Master device. Figure 8 demonstrates results for a 2 and 4 lb weights. Subjects tended to push up (opposing gravity) with their unaffected arm as well, thus reducing the force required by the Slave arm, in particular for larger weights. Such a force drift can be observed in Figure 8B.

### Motor function assessment data (FMA and WMFT)

Changes in both FMA and WMFT between pre- and post-assessments are reported in Table 4. The mean changes in FMA and WMFT were 15.2 and 19.1%, respectively. No significant differences were found. A *t*-test for the FMA and WMFT, with α = 0.05, resulted in *p*_FMA_ = 0.66 and *p*_WMFT_ = 0.75, respectively.

### Proprioception assessment data

**Figure 9:**
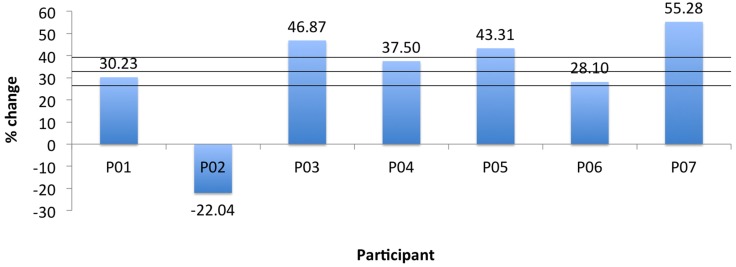
**Robot proprioception change for all participants**. Horizontal lines indicate mean and ±1 SD.

**Figure 10:**
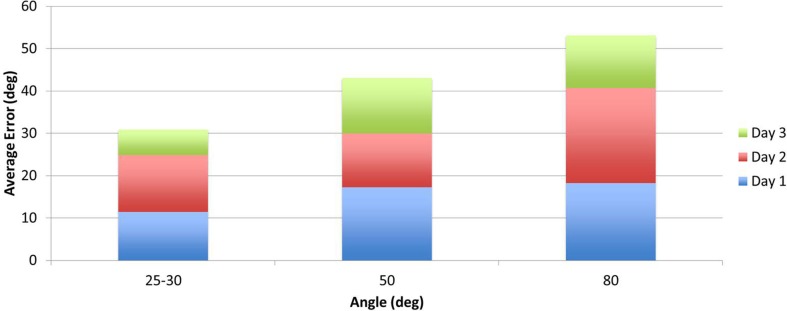
**Participants’ proprioception test mean daily errors per assessment angle**.

Two measurements per angle (30°, 50°, 80°) were collected daily and averaged. Daily errors were then calculated by summation of the angle errors. The mean change in the proprioception error measurement, pre- to post-assessment, was 32.8% as shown in Table 4 with a SD of 6.3°, and was found statistically significant with α = 0.05 and *p*_prop_ = 0.033. Participant P02 had significantly larger errors in the second measurement of day 3. Repeating the above calculations and including only the first measurement for P02 results in a mean of 41.1% with a SD of 5° and *p*_prop_ = 0.0044. Further, the participants’ error changes in percentage from day 1 to day 3 are shown Figure 9. For P02, if considering only the first measurement then instead of a −22.04% error increase, an error reduction change of 38.1% is calculated. Mean errors per angle over the three training days are shown in Figure 10. The daily cumulative errors at the 80° angle were the greatest. The errors in day 3 were smaller than the first day of testing for all angles and smaller than the second day of testing for the 30 and 80° angles.

**Figure 11:**
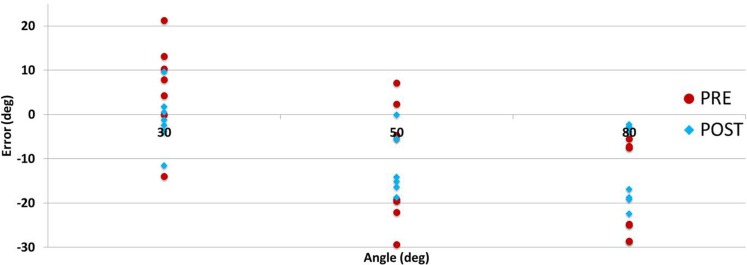
**Participants’ proprioception test directional errors per angle pre- (day 1) and post- (day 3) training**.

Directional errors were analyzed observing for under/over shooting biases in Figure 11. There were approximately four times more undershooting instances than overshooting (95:25), and increasingly more cases as the testing angle was larger. Other researchers have reported directional errors with varying biases (Adamovich, 1998; Goble and Brown, 2008; Leibowitz et al., 2008; Fuentes and Bastian, 2010). It should be noted that there were significant differences in the studies methods (arm position, orientation, movement direction, etc.) and populations making it challenging to draw conclusions.

## Discussion

We developed the BWRD to engage users in a series of customized self-driven exercises. Specifically, we examined the feasibility of methods that implement impairment force reflection on the contralateral arm. This is an important study that blends robotics with exercise. Our BWRD provided haptic feedback to encourage the stroke survivors to control the movements of their arms – a central component in stroke rehabilitation and initiating neural plasticity. The acquired results offer important insights toward the potential use of bilateral robotic rehabilitation and its therapeutic implications in individuals with stroke.

Although it was an intensive 3 day feasibility study, it was still considered short as compared to clinical rehabilitation trials (Cauraugh et al., 2010; Van Delden et al., 2012), thus the FMA, WMFT, and proprioception results cannot be interpreted assertively. In the future, a longer duration study will be designed in order to determine the clinical significance of the results using the BWRD in terms of progression of motor function and proprioception. However, the positive change in the scores of FMA and WMFT are encouraging and suggesting that training with BWRD may contribute toward motor recovery.

On the other hand, the proprioception results may signify the viability of the system in assessing proprioception. The proprioceptive assessments show a reduction in angular error that was deemed statistically significant. All the second angle measurements for P02 taken at day 3 have large errors that may be considered as outliers. Consequently, neglecting measurement two for this participant improves the results. An apparent result emerging from the proprioception test is revealed in having greater errors at the 80° angle compared to the 50 and 30° target angles tested. One interpretation is that traveling to a further target results in a larger error; similar findings had been reported in the literature (Lönn and Crenshaw, 2000). The participants had a tendency to flex their shoulder when matching angles, more so in larger angles (they were instructed of this and encouraged to make a correction). This may have contributed to increased errors in the 80° angle assessments, possibly due to matching forearm orientation rather than the elbow joint angle. The proprioception test was carried out while the target arm was passive, eliminating the need for memorization of its location (Goble, 2010). The target arm was positioned manually and as a consequence may have triggered the Tau effect (Helson, 1930), whereby the time to target may influence the participants judgment. Future work should formalize the target reaching procedure. Additionally, passive target positioning has been shown to result in larger errors compared to active positioning (Fuentes and Bastian, 2010). Participant P06 demonstrated the least amount of WMFT improvement. This was expected, as his baseline FMA score indicated minor motor impairment. This participant has left neglect and was considered to have trouble with proprioception in activities of daily living (ADL). Despite the pre-assessment not showing greater proprioception deficiency compared to the rest of the participants, his test results show a smaller error reduction relative to the other participants (excluding P02 second measurement, which is thought to be an outlier). It is important to consider that in contralateral matching, it may be challenging to discern between errors arising from the target arm or the acting arm (Goble, 2010).

Comparing the performance among tasks #1–3 may offer insights as to the specifics of the arm impairment. Difficulty in completing task #1 may suggest residual arm tension. A similar case can be seen in Figure 5D where the affected arm force vanishes in the arm extension component of the movement, suggesting that the participant was unable to relax and weigh down the affected arm. Further, a more severe case may display an opposing force where the affected arm flexes rather than extends. Trouble in task #3 may indicate a case of muscle atrophy evident by a low force in either the extension or flexion components of the movement (Ada, 2003). Accordingly, Figure 5F may demonstrate a case of extensor muscle weakness. Struggling with task #2 may point to either of the aforementioned conditions or alternatively to a proprioceptive deficiency. As mentioned, in task #2 the affected arm should track the unaffected arm. However, a lacking estimation of the affected arm position could result in increased interaction forces.

In task #4 (Figure 6), the healthy arm performed a force-feedback search of the position of the affected arm. This exercise may be implemented such that the affected arm performs a search, of the unaffected arm, with variation of the maximum resistance based on the individual’s impairment level. In task #4, the user must also focus on sensory input, in addition to performing muscle exercises, potentially enhancing rehab effectiveness.

Few subjects found task #5 challenging to accomplish, resulting in the forces differing considerably. This is likely due to the coordination complexity involved in applying equal forces simultaneously with both limbs in a dynamic task. An approach suggested to participants was to fix the force applied with the Master arm, and force-explore with the Slave arm. By doing so, the subject was able to decompose and simplify the task. It is worth noting, other than the subjects own somatosensory input, no external feedback was provided as to which arm is applying a higher or lower force. Consequently, subjects often tried increasing the force with the arm that was applying the greater force in the first place. The arm applying the greater forces was often the unaffected arm. Those subjects, who struggled with the task the most, were offered a visual feedback of the forces applied by each arm in the form of a graphical tank indicator, however, with equivocal benefit. A visual indicator seemed to distract participants from focusing on other sensory feedback. Nevertheless, it is possible that this task may be improved by providing auxiliary feedback such that the user can infer either arm force state. Task #5 can be adjusted to gradually be more difficult as the subject’s arm function improves by reducing the force difference required (set to 1 Nm in this study).

Task #6 can also be designed with additional complexities as well as be implemented as a dynamic task. In future work, we conceive having the subject apply the Master arm force independently (i.e., without using weights), which would increase the coordination and skill required. Additionally, the task may be expanded to a dynamic case where, while opposing each other, the arms are simultaneously moved along the ROM. These changes to the task will increase the difficulty level for subjects who have made enough progress with the more simple structure of task #6. It is interesting to note that a dynamic case of task #6 may appear to be similar to task #3. There is a fundamental difference, however, in the task underlying workings; whereas in task #3 the Master arm is driving position and encountering resistance (an impedance system); in task #6, both arms are driving a force resulting in a position change (an admittance system). The difference between task #3 and #6 may have implications to the user task planning and motor learning.

There are several recognized robotic therapy modalities often implemented in the research and industry settings. These generally include passive, assistive, assist as needed (AAN) (similar to active assistance), resist as needed (RAN) (similar to active constrained), and resistive training, along some other variations of these (Wang, 2013; Basteris et al., 2014), and can be operated in unilateral or bimanual systems (Li et al., 2011; Van Delden et al., 2012). The training protocol in this work shares similarities to some of the aforementioned common therapy approaches. In the most basic interpretation, tasks #1–3 can be considered as a passive-mirrored modality toward the impaired arm (driven by the contralateral arm). In addition, task #1 may be regarded as resistive and task #2 as RAN toward the intact arm. The modality in task #3 can be considered as resistive for both arms, with the resistance being initiated by the user. Task #5 may also be considered as a RAN modality toward both arms, with the resistance generated if the participant is deviating from the required force difference. Tasks #4 and #6 are fundamentally resistive tasks. In developing the BWRD, we wanted to design a system that would require the user’s attention and active engagement in the difficulty setting and performance of the training. There is evidence suggesting that the patient’s active involvement in the training contributes to motor improvement (Basteris et al., 2014).

Published works concerning development of rehab robots that give the user a sensation or a form of physical feedback of the impairment, to the contralateral limb, are sparse (Johnson et al., 2005; Rashedi et al., 2009; Li et al., 2013). This work is targeted at supplementing to this research field with a unique bimanual system and a novel training protocol.

We report several variables that may have potentially limited our results. Our participants were all males, which may have generated an undesired bias. We noticed a learning curve for participants using the BWRD. Several trials were required before users comprehended the system operation and how they could control it. We expect that the changes in the clinical assessment results are in part due to this familiarity with the system. Our participants were all right handed with left hand affected by stroke, except participant P01. It would be instructive to include and compare the results and rate of improvement with individuals having the preferred arm affected as studies show that the non-preferred arm tends to perform better in proprioception tasks (Bagesteiro and Sainburg, 2003; Goble et al., 2006). Device fit variability between sessions, and contact sensory feedback between the arm and the BWRD likely also played a role in position accuracy. One participant appeared to augment their sensation and arm localization by pushing against the Slave arm while performing the proprioception position matching. In task #4, subjects were asked to concentrate and respond to the haptic feedback; they had nevertheless access to a visual feedback, potentially introducing biases by utilization of non-proprioceptive afferent circuitry. On the other hand, offering a variety of feedback information can enhance the internal motor control model, and consequently task proficiency.

The results of this preliminary study are promising, demonstrating that the participants were able to adapt to the system and complete the protocol with varied success levels. Future tests involving larger populations and added training sessions are needed to elucidate the system’s rehabilitation potential and to distinguish between motor control improvement and simply task familiarity through practice. The scope of this study, however, was aimed at demonstrating system potential and feasibility, making such inferences inappropriate. Future work would explore enabling additional upper-limb joints (i.e., more DOF). The authors also recognize opportunities of expanding the protocol in a manner conducive to gaining quantifiable measures of the task performance and hence the subjects’ conditions. One way to achieve this is by obtaining and utilizing kinematic data, such as movement time and peak velocity, as other authors have suggested (Wu et al., 2011).

## Conclusion

Rehabilitation is a key process to recovery for individuals affected by stroke. This study investigated a new bimanual system and protocol involving the use of robotic training, with haptic feedback, offering subjects the opportunity to sense their own deficiency level and improve through this process. We implemented a 3-day study to evaluate the adoption by participants and potential of the system to contribute to rehabilitation. All participants were able to complete our protocol tasks, some with good success. One of our constructive results is a significant error decrease in the proprioception measure, although this result comes with a caveat due to only a week-long training time period. Based on the collected results, we are encouraged to proceed with a follow-up investigation. We suggest that innovative bimanual tasks summoning the participants’ skill and concentration as well as autonomy in setting the difficulty level, can play a key role in stimulating rehabilitation.

## Conflict of Interest Statement

The authors declare that the research was conducted in the absence of any commercial or financial relationships that could be construed as a potential conflict of interest.
